# Reactions of glycidyl derivatives with ambident nucleophiles; part 2: amino acid derivatives

**DOI:** 10.1186/1860-5397-3-28

**Published:** 2007-09-27

**Authors:** Gerald Dyker, Andreas Thöne, Gerald Henkel

**Affiliations:** 1 Dept. of Chemistry & Biochemistry, Ruhr-University Bochum, Universitätsstrasse 150, D-44780 Bochum, Germany; 2 Dept. of Chemistry, Paderborn University, 33098 Paderborn, Germany

## Abstract

A three-step procedure for the synthesis of multifunctionalized heterocycles from a pyroglutamic acid derivative, glycidyl components and anilines by nucleophilic substitution and cobalt catalysis is presented.

## Introduction

Recently we investigated the formation of 5- and 6-membered heterocycles from ethyl acetoacetate and glycidyl derivatives such as epichlorohydrin.[1] We found that the selectivity of this reaction was strongly influenced by the solvent and by the base applied, and especially by the nature of the leaving group at the glycidyl derivative. Despite the preparative potential of analogous cyclization reactions with other ambident nucleophiles, reports on this subject are surprisingly rare.[2]

**Scheme 1:**
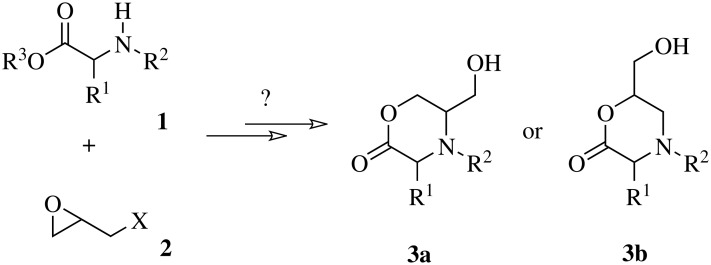
Planned construction for morpholinones **3** from amino acid and glycidyl derivatives **1** and **2**. R^1^, R^3^ = H, Alkyl, Aryl; R^2^ = H, Acyl; X = leaving group: Cl, Br, I, *p*-OTs.

For instance, amino acids and their derivatives **1** should be suitable as ambident nucleophiles. In principle the reaction with glycidyl derivatives **2** should lead to morpholinones **3** as depicted in Scheme 1, a class of heterocycles that are interesting as a crucial moiety of drugs for the treatment of various inflammatory and other diseases. [3–5]

## Results and Discussion

A high selectivity for the formation of one of the regioisomers **3a** and **3b** is anticipated, depending on the initial nucleophilic substitution either by the carboxylic acid or by the amino functionality, in both cases at a terminal carbon atom of **2**.

All attempts to achieve a one-step cyclization according to Scheme 1 starting from glycine or from phenylglycine seemed to fail under various reaction conditions (for instance one equiv. of NaOH, K_2_CO_3_ or triethylamine in water), regularly giving rise to solid, presumably oligomeric material, insoluble even in DMSO.

We therefore tested the stepwise, controlled synthesis of products of type **3a** with glycidyl esters of N-acyl [6] and N-tosyl glycine and phenylglycine as isolated key intermediates: under basic reaction conditions (NaH, *sec*-BuLi or LDA in THF, or potassium carbonate in DMF) these glycidyl esters gave no defined cyclization products, although related reactions have some precedent (for instance cyclization of urethanes of glycidol).[7]

**Scheme 2:**
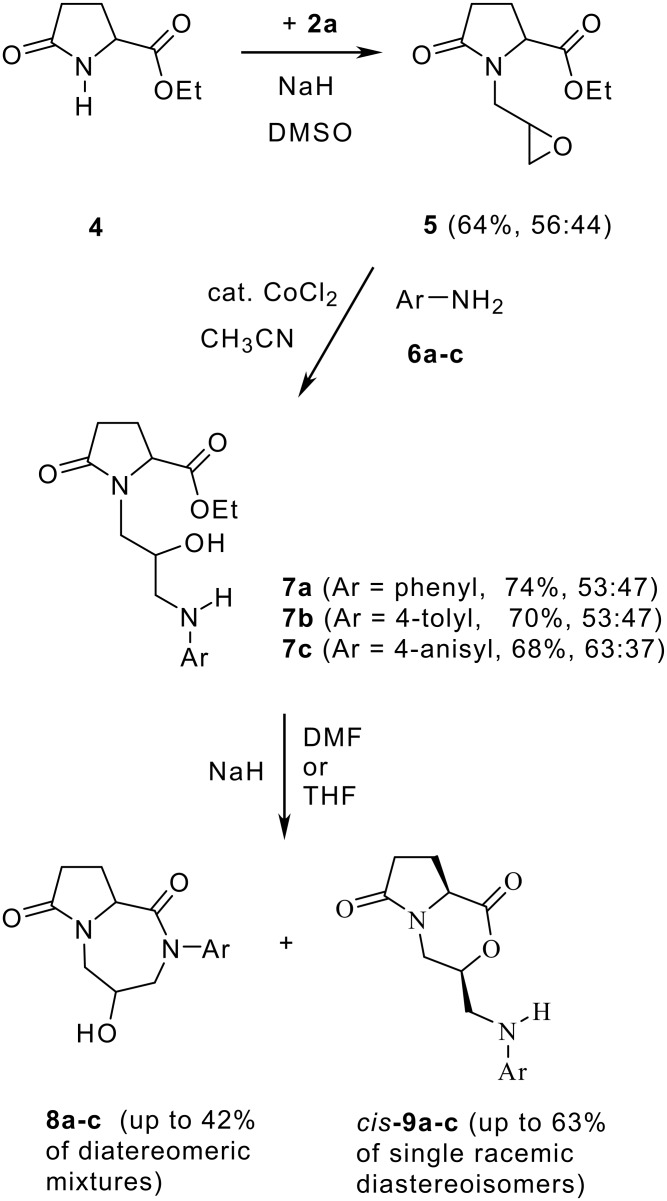
Synthesis of pyrrolidinone-fused heterocycles from a glycidyl substituted pyroglutamic acid ester **5** (ratio of diastereoisomers determined by ^1^H NMR spectroscopy).

For the synthesis of products of type **3b** we decided to use the pyroglutamic acid derivative **5** as model compound (Scheme 2, see Supporting Information File 1 for full experimental data), readily accessible as a mixture of diastereoisomers by N-alkylation of racemic **4** [8] with epibromohydrin (**2a**) in DMSO. Again, under basic as well as acidic conditions (for instance heating in trifluoroacetic acid) no cyclization products were obtained. Finally we succeeded in achieving a cyclization by a two-step procedure: the cobalt-catalyzed addition of electron-rich anilines **6** according to Iqbal [9] let to amino alcohols **7**, which then cyclized in basic medium (NaH in DMF or THF), either to give 7-membered lactams **8** or 6-membered lactones **9** (Table 1). Most interestingly, the lactones **9** were always found as a single diastereoisomer, while for the lactams **8** various ratios of the diastereoisomers were detected (ratio ranging from 7:3 to 4:6, measured by ^1^H NMR).

**Table 1:** Base-induced cyclization of **7**.

entry	cond.^a^	Ar Yield [%]:	**8**	**9**	**10**

1	A	**a** (phenyl)	22	63	-
2	B	**a** (phenyl)	13	42	-
3	C	**a** (phenyl)	8	60	28
4	A	**b** (4-tolyl)	42	36	-
5	B	**b** (4-tolyl)	9	60	-
6	C	**b** (4-tolyl)	30	36	9
7	A	**c** (4-anisyl)	36	10	-
8	B	**c** (4-anisyl)	25	50	-

Isolated yields are given. a) Reaction conditions: 1 equiv. NaH, 90 min room temperature, concentration ~30 mmol/L; A: in THF, B: in DMF, C: in DMF in the presence of air.

**Figure 1:**
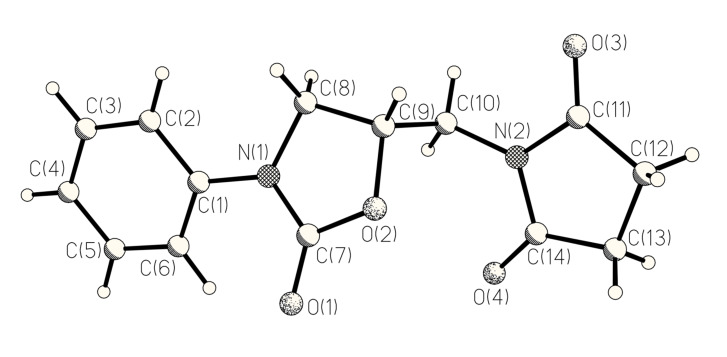
Structure of oxidation product **10a** in the crystal.

**Scheme 3:**
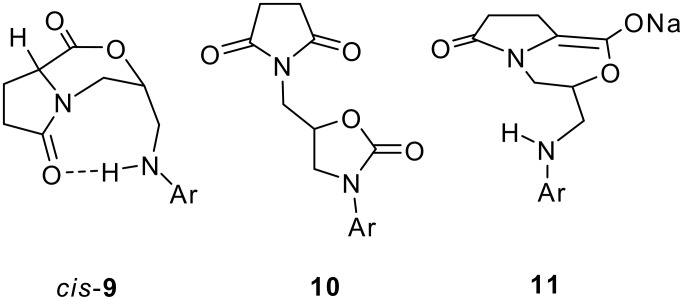
Special product conformers, by-products and intermediates of the transformation of **7**.

Under the strongly basic reaction conditions of the cyclization process the α-proton of the amino acid moiety is obviously acidic enough to allow epimerization at this centre. This is in accord with the exclusive formation of a single diastereoisomer of **9** and with the occurrence of **10a** as a byproduct in up to 28 % yield, when the reaction was carried out in the presence of air (for its X-ray crystal structure see Figure 1; X-ray data have been deposited at the Cambridge Crystallographic Data Centre; deposition number CCDC-214083; Copies of the data can be obtained free of charge on application to CCDC, 12 Union Road, Cambridge CB2 1EZ); clearly **10a** is an oxidation product of an intermediary enolate, such as **11** (Scheme 3), which is of course also the key intermediate for the diastereoselective formation of **9**. We assume, that *cis*-**9** with the *endo*-oriented aminomethyl group is the preferred diastereoisomer, which profits thermodynamically from a weak intramolecular hydrogen bond. According to ab initio calculations for *cis*-**9a**, (B3LYP, 6-31G*, zero point energy included) this structure is indeed 1.9 kcal/mol more stable than its *exo*-oriented conformer and 2.2 kcal/mol more stable than its *trans*-stereoisomer.

## Conclusion

In summary, we achieved the annulation reaction of amino acid derivatives with glycidyl compounds as functionalized C_3_ building blocks in combination with a cobalt-catalyzed addition of anilines to the epoxide functionality. This rather selective transition-metal catalysed step builds up the alcohol and the amino functionality, which compete in the final ring closure. On the other hand we found it surprising, that the rather simple, straight forward looking reaction in Scheme 1 could not be realized directly. Clearly, a lot more experimental results have to be collected before the reactivity of these classical substrates will be sufficiently understood.

## Supporting Information

File 1 Experimental section. File 1 for full experimental data.
