# Supplementary material for: Reactions of glycidyl derivatives with ambident nucleophiles; part 2: amino acid derivatives
Source: Beilstein J Org Chem. 2007 Sep 27;3:28. doi: 10.1186/1860-5397-3-28 (PMC2151071; doi:10.1186/1860-5397-3-28)
Supplement: File 1 — Experimental section. File 1 for full experimental data. [file Beilstein_J_Org_Chem-03-28-s001.doc]

Experimental section for:

###### Reactions of glycidyl derivatives with ambident nucleophiles;

###### part 2: amino acid derivatives

Gerald Dyker^1^, Andreas Thöne^1^, and Gerald Henkel^2^

^1^Dept. of Chemistry & Biochemistry, Ruhr-University Bochum, Universitätsstrasse 150, D-44780 Bochum, Germany

^2^Dept. of Chemistry, Paderborn University, 33098 Paderborn, Germany

Experimental Section

**General Remarks:** m.p. (uncorrected): Reichert Thermovar. IR: Perkin Elmer 841 and 983. NMR: Bruker DPX-200, WM-300, DRX-400, AM-500; ^1^H NMR spectra were recorded in CDCl_3_ or in DMSO-D_6_ with TMS as the internal standard; ^13^C NMR spectra were generally measured by using CDCl_3_ as the solvent and the internal standard. In the case of diastereoisomeric mixtures the ^1^H NMR and ^13^C NMR signals of each diastereoisomer were tracked by HH- and CH-COSY spectra. MS: MAT 700 ITD (70 eV) and Varian MAT 311 A. For analytical TLC precoated plastic sheets “POLYGRAM SIL G/UV254” from “Macherey-Nagel” were used. EA: Elementar/Hanau Vario EL.

**N-(Oxiranylmethyl)-pyrrolidin-2-on-5-carboxylic acid ethyl ester (****5) :** To a solution of 400 mg (2.54 mmol) of pyroglutamic acid ethyl ester **4** in 30 ml of dry DMSO at 10 °C 102 mg (2.54 mmol) of NaH (60% in mineral oil) were added under nitrogen. After 30 min stirring 0.220 ml (2.54 mmol) of epibromohydrin **2** (X = Br) were added to the suspension and stirring was continued for 19 h at room temperature. Following hydrolysis with 100 ml of water the mixture was extracted three times with 30 ml of dichloromethane and the combine organic layer was concentrated. The residual oil was distilled at the Kugelrohr oven at 160 °C/0.03 mbar: 346 mg (64%) of cyclic amide **5** as colourless oil (mixture of diastereoisomers A and B in the ratio 56:44 according to ^1^H NMR). - IR (KBr): $\tilde{\nu}$ = 2983 cm^-1^ (m), 2936 (m), 2334 (m), 1738 (s), 1697 (s), 1443 (m), 1413 (s), 1372 (m), 1335 (m), 1259 (s), 1196 (s), 1153 (m), 1116 (w), 1097 (w), 1029 (m), 958 (w), 912 (w), 912 (w), 954 (m), 756 (w). ^1^H NMR (CDCl_3_, 500 MHz): mixture of diastereoisomers in the ratio 56:44; diastereoisomer A: δ = 1.29 ppm (t, *J* = 5.0 Hz, 3H), 2.07-2.11 (m, 1H), 2.34-2.53 (m, 3H), 2.49-2.53 (m, 2H), 2.70 (dd, *J* = 14.8, 8.2 Hz, 1H), 2.75-2.78 (m, 1H), 3.05 (m, 1H), 4.22 (q, *J* = 5.0 Hz, 2H), 4.44 (m, 1H); diastereoisomer B: δ = 1.31 ppm (t, *J* = 5.0 Hz, 3H), 2.07-2.11 (m, 1H), 2.34-2.53 (m, 3H), 2.49-2.53 (m, 2H), 2.75-2.78 (m, 1H), 3.09 (m, 1H), 3.47 (dd, *J* = 15.0, 5.2 Hz, 1H), 3.65 (dd, *J* = 15.0, 3.2 Hz, 1H), 4.22 (m, 3H). ^13^C NMR (CDCl_3_, 125.8 MHz): diastereoisomer A: δ = 14.14 ppm (q), 23.17 (t, C-3), 29.18 (t, C-4), 44.68 (t), 44.69 (t), 50.29 (d, C-7), 60.04 (d, C-2), 61.49 (t), 171.77 (s), 175.29 (s, C-5); diastereoisomer B: δ = 14.14 ppm (q), 23.43 (t, C-3), 29.05 (t, C-4), 43.40 (t, C-6), 45.16 (t, C-8), 49.75 (d, C-7), 60.81 (d, C-2), 61.59 (t), 172.05 (s), 175.61 (s, C-5). MS (EI, 70 eV, 135 °C): *m/z* (%) = 213 (8) [M+], 141 (9), 140 (100), 110 (21), 98 (28), 96 (9), 84 (22), 75 (8), 68 (8), 56 (18), 41 (17). Elemental analysis calcd for C_10_H_15_NO_4_ (213.23): C 56.33, H 7.09, N 6.57; found: C 56.33, H 7.02, N 6.49.

**1-(2-Hydroxy-3-phenylamino-propyl)-5-oxo-pyrrolidine-2-carboxylic acid ethyl ester (7a):** A mixture of 1.65 g (7.74 mmol) of epoxide **4** (diastereoisomers 56:44), 0.704 ml (7.74 mmol) of aniline and 100 mg (0.774 mmol) of dry CoCl_2_ in 30 ml of dry acetonitrile was stirred at room temperature in a screw-capped tube for 3 d. The solvent was removed in vaccuo (50 °C, 200 mbar to 0.02 mbar) and the residue was purified by flash chromatography; TLC (silica/ethyl acetate): Rf = 0.85 (aniline), 0.39 (**7a**). Yield: 1.76 g (74%) of a 53:47 mixture of diastereoisomers of **7a** as yellow oil. - IR (KBr): $\tilde{\nu}$ = 3350 cm^-1^ (w, br, NH), 2929 (w), 2335 (w), 2295 (w), 1738 (s, C=O), 1693 (s, C=O), 1603 (w), 1500 (w), 1416 (w), 1377 (w), 1203 (m), 1099 (w), 1031 (w), 696 (w). ^1^H NMR (CDCl_3_, 500 MHz): mixture of diastereoisomers in the ratio 53:47; diastereoisomer A: δ = 1.28 ppm (t, *J* = 7.2 Hz, 3H), 2.09 (m, 1 H), 2.39 (m, 2 H), 2.52 (m, 1 H), 3.13 ("dd", "J" = 14.5, 6.9 Hz, 1 H), 3.22 ("dd", "J" = 14.5, 4.9 Hz, 1 H), 3.27 (dd, "J" = 14.6, 3.1 Hz, 1 H), 3.53 (dd, *J* = 14.5 Hz, 3.0 Hz, 1 H), 4.05 (m, 1 H), 4.22 (m, 2 H, OCH2), 4.32 (m, 1 H), 6.65 (m, 2 H), 6.71 (m, 1 H), 7.16 (m, 2 H); diastereoisomer B: δ = 1.28 ppm (t, *J* = 7.2 Hz, 3 H), 2.09 (m, 1 H), 2.39 (m, 2 H), 2.52 (m, 1 H), 3.10 ("dd", *"J"* = 14.5 Hz, 6.8 Hz, 1 H), 3.24 ("dd", *"J"* = 10.0 Hz, 5.0 Hz, 1 H), 3.35 (dd, *J* = 14.5 Hz, 7.6 Hz, 1 H), 3.64 (dd, *J* = 14.5 Hz, 7.1 Hz, 1 H), 4.05 (m, 1 H), 4.22 (m, 2 H)*,* 4.32 (m, 1 H), 6.65 (m, 2 H), 6.71 (m, 1 H), 7.16 (m, 2 H). ^13^C NMR (CDCl_3_, 125.8 MHz): diastereoisomer A: δ = 14.07 ppm (q), 23.52 (t), 29.33 (t), 47.54 (t), 47.77 (t), 61.71 (t), 61.98 (d), 69.17 (d), 113.18 (d), 117.72 (d), 129.19 (d), 148.11 (s), 172.28 (s), 176.95 (s); diastereoisomer B: δ = 14.07 ppm (q), 23.41 (t), 29.33 (t), 47.02 (t), 47.34 (t), 61.37 (d), 61.69 (t), 68.38 (d), 113.15 (d), 117.62 (d), 129.19 (d), 148.11 (s), 172.18 (s), 177.01 (s). MS (EI, 70 eV, 145 °C): *m/z* (%) = 306 (3) [M+], 200 (15), 84 (15), 78 (27), 76 (81), 71 (19), 170 (21), 158 (19), 142 (24), 140 (10), 126 (16), 124 (8), 106 (20), 98 (39), 96 (10), 5 (8), 84 (100), 68 (20), 56 (11), 55 (8), 41 (35). HRMS failed due to the weak signal of the molecular ion.

**1-(2-Hydroxy-3-(4-methylphenylamino)-propyl)-5-oxo-pyrrolidine-2-carboxylic acid ethyl ester (7b):** A mixture of 1.01 g (4.75 mmol) of epoxide **4** (diastereoisomers 56:44), 508 mg (4.75 mmol) of toluidine and 61.6 mg (0.475 mmol) of dry CoCl_2_ in 25 ml of dry acetonitrile was stirred at room temperature in a screw-capped tube for 3 d. The solvent was removed in vaccuo (50 °C, 200 mbar to 0.02 mbar) and the residue was purified by flash chromatography; TLC (silica/ethyl acetate): Rf = 0.86 (toluidine), 0.48 (**7b**). Yield: 1.07 g (70%) of a 53:47 mixture of diastereoisomers of **7b** as a yellow and sticky oil. - IR (KBr): $\tilde{\nu}$ = 3373 cm^-1^ (m, br, NH), 2923 (m), 1739 (s, C=O), 1672 (s, C=O), 1615 (m), 1520 (m), 1450 (m), 1416 (m), 1192 (m), 1106 (m), 1046 (m), 811 (m). ^1^H NMR (CDCl_3_, 500 MHz): mixture of diastereoisomers in the ratio 53:47; diastereoisomer A: δ = 1.28 ppm (t, *J* = 7.2 Hz, 3H), 2.09 (m, 1H), 2.23 (s, 3H), 2.39 (m, 2H), 2.51 (m, 1H), 3.07 (dd, J= 12.7, 7.1 Hz, 1H), 3.18 (dd, J= 12.8, 5.0 Hz, 1H), 3.30 (dd, *J* = 14.4 Hz, 7.6 Hz, 1H), 3.62 (dd, *J* = 14.5, 7.2 Hz, 1H), 4.03 (m, 1H), 4.22 (m, 2H), 4.34 (m, 1H), 6.57 ( dd, *J* = 8.4, 1.9 Hz, 1H), 6.97 ( d, *J=* 8.0 Hz, 2H); diastereoisomer B: δ = 1.28 ppm (t, *J* = 7.2 Hz, 3H), 2.09 (m, 1H), 2.23 (s, 3H), 2.39 (m, 2H), 2.51 (m, 1H), 3.09 (dd, *J* = 13.0 Hz, 6.8 Hz, 1H), 3.20 (dd, *J* = 12.8, 4.6 Hz, 1H), 3.25 (dd, *J* = 14.5, 2.8 Hz, 1H), 3.62 (dd, *J* = 14.5, 7.2 Hz, 1H), 4.03 (m, 1H), 4.22 (m, 2H), 4.34 (m, 1H), 6.57 (dd, *J* = 8.4, 1.9 Hz, 2H), 6.97 (d, *J* = 8.0 Hz, 2H). ^13^C NMR (CDCl_3_, 125.8 MHz): diastereoisomer A: δ = 14.10 ppm (q), 20.33 (q), 23.56 (t), 29.36 (t), 47.77 (t), 48.01 (t), 61.72 (t), 61.97 (d), 69.27 (d), 113.48 (d), 127.09 (s), 129.70 (d), 145.82 (s), 172.29 (s, C=O), 176.91 (s, C=O); diastereoisomer B: δ = 14.10 ppm (q), 20.33 (q), 23.47 (t), 29.26 (t), 47.12 (t), 47.83 (t), 61.41 (d), 61.69 (t), 68.51 (d), 113.44 (d), 126.98 (s), 129.70 (d), 145.82 (s), 172.21 (s, C=O), 177.06 (s, C=O). MS (EI, 70 eV, 155 °C): *m/z* (%) = 320 (16) [M+], 302 (8), 274 (6), 200 (8), 158 (18), 146 (7), 145 (7), 126 (10), 121 (27), 120 (100), 107 (7), 98 (6), 91 (16), 84 (15), 45 (7), 41 (7). HRMS: calcd for C_17_H_24_N_2_O_4_: 320.17362; found 320.17384.

**1-(2-Hydroxy-3-(4-methoxyphenylamino)-propyl)-5-oxo-pyrrolidine-2-carboxylic acid ethyl ester (7c):** A mixture of 440 mg (2.06 mmol) of epoxide **4** (diastereoisomers 56:44), 254 mg (2.06 mmol) of anisidine and 26.8 mg (0.206 mmol) of dry CoCl_2_ in 25 ml of dry acetonitrile was stirred at room temperature in a screw-capped tube for 3 d. The solvent was removed in vaccuo (50 °C, 200 mbar to 0.02 mbar) and the residue was purified by flash chromatography; TLC (silica/ethyl acetate): Rf = 0.82 (anisidine), 0.30 (**7c**). Yield: 471 mg (68%) of a 63:73 mixture of diastereoisomers of **7c** as a brownish and sticky oil. - IR (KBr): $\tilde{\nu}$ = 3371 cm^-1^ (w, br, NH), 2923 (w), 1738 (s, C=O), 1674 (s, C=O), 1512 (s), 1458 (w), 1414 (w), 1235 (s), 1188 (s), 1102 (w), 1074 (m). ^1^H NMR (CDCl_3_, 500 MHz): mixture of diastereoisomers in the ratio 53:47; diastereoisomer A: δ = 1.29 ppm (t, *J* = 7.1 Hz, 3H), 2.10 (m, 1H), 2.39 (m, 2H), 2.53 (m, 1H), 3.04 (dd, *J* = 12.6, 7.2 Hz, 1H), 3.18 (dd, *J* = 12.6, 4.5 Hz, 1H), 3.33 (dd, *J* = 14.5, 7.6 Hz, 1H), 3.55 (dd, *J* = 14.5, 3.0 Hz, 1H), 4.00 (m, 1H), 4.22 (q, *J* = 7.1 Hz, 2H), 4.32 (m, 1H), 6.62 (dd, *J* = 9.0, 1.4 Hz, 2H), 6.77 (dd, *J* = 6.7, 2.3 Hz, 2H); diastereoisomer B: δ = 1.29 ppm (t, *J* = 7.1 Hz, 3H), 2.10 (m, 1H), 2.39 (m, 1H), 2.53 (m, 1H), 3.07 (dd, *J* = 12.6, 6.9 Hz, 1H), 3.16 (dd, *J* = 12.8, 4.8 Hz, 1H), 3.28 (dd, *J* = 14.6, 3.2 Hz, 1H), 3.62 (dd, *J* = 14.5, 7.2 Hz, 1H), 4.00 (m, 1H), 4.22 (q, *J* = 7.1 Hz, 2H), 4.32 (m, 1H), 6.62 (dd, J = 9.0, 1.4 Hz, 2H), 6.77 (dd , J = 6.7, 2.3 Hz, 2H). ^13^C NMR (CDCl_3_, 125.8 MHz): diastereoisomer A: δ = 14.13 ppm (q), 23.60 (t), 29.39 (t), 47.24 (t), 48.97 (t), 55.76 (q), 61.75 (t), 62.00 (d), 69.20 (d), 114.87 (d), 115.07 (d), 141.88 (s), 152.71 (s), 172.32 (s), 176.96 (s, C=O); diastereoisomer B: δ = 14.13 ppm (q), 23.52 (t), 29.39 (t), 47.81 (t), 48.87 (t), 55.76 (q), 61.50 (d), 61.75 (t), 68.51 (d), 114.87 (d), 115.07 (d), 141.86 (s), 152.67 (s), 172.26 (s), 177.14 (s, C=O). MS (EI, 70 eV, 180 °C): *m/z* (%) = 337 (3), 336 (16) [M+], 290 (10), 200 (7), 158 (24), 137 (18), 136 (100), 126 (11), 108 (9), 98 (19), 84 (22), 68 (9), 45 (9), 41 (13). HRMS: calcd for C_17_H_24_N_2_O_5_: 336.16853; found 336.16802.

**Base-induced cyclization of 7a (Table 1, entry 3):** 220 mg (0.718 mmol) of **7a** in 20 ml of DMF were treated with 29 mg (0.725 mmol) of sodium hydride (60% in mineral oil) for 1.5 h at room temperature in the presence of air. After addition of 1 equiv. of NaHPO_4_/water the mixture was concentrated at 50 °C/<1 mbar. The residue was fractionated by flash chromatography (silica, ethyl acetate as first eluent, ethanol as second). **1^st^ Fraction** (silica/ethyl acetate; R_f_ = 0.53): 55 mg (28%) of 1-(2-oxo-3-phenyloxazolidin-5-ylmethyl)-pyrrolidine-2,5-dione **10a** as colourless solid with mp 165 °C. - IR (KBr): $\tilde{\nu}$ = 1994 cm^-1^ (w), 1744 (s, C=O), 1699 (s, C=O), 1600 (w), 1418 (rn), 1403 (m), 1320 (m), 1232 (m), 1179 (m), 1137 (m), 1118 (w), 1080 (w), 1009 (w), 977 (w), 977 (w), 759 (w). ^1^H NMR (CDCl_3_, 500 MHz): δ = 2.77 ppm (s, 4H), 3.75 (dd, *J* = 13.9, 4.9 Hz, 1H), 3.82 (dd, *J* = 9.1, 5.9 Hz, 1H), 4.01 (dd, *J* = 13.9, 7.5 Hz, 1H), 4.11 (m, 1H), 4.92 (m, 1H), 7.16 (t, *J* = 7.4, 1H), 7.37 (dd, J= 8.7, 7.4 Hz, 2H), 7.51 (dd, J = 8.8, 1.1 Hz, 2H). ^13^C NMR (CDCl_3_, 125.8 MHz): δ = 28.18 ppm (t), 41.71 (t), 48.41 (t), 68.90 (d), 118.41 (d), 124.43 (d), 129.17 (d), 137.87 (s), 153.88 (s), 176.85 (s). MS (EI, 70 eV, 190 °C): *m/z* (%) = 275 (8), 274 (44) [M+], 230 (37), 162 (14), 138 (16), 132 (25), 131 (78), 130 (100), 119 (10), 118 (33), 106 (24), 105 (23), 104 (41), 91 (19), 84 (9), 77 (53), 56 (14), 55 (21), 51 (15). Elemental analysis calcd for C_14_H_14_N_2_O_4_ (274.28): C 61.31, H 5.15, N 10.21; found: C 61.24, H 5.22, N 10.16. **2^nd^ Fraction** (silica/ethyl acetate; R_f_ = 0.18): 15 mg (8%) of 4-hydroxy-2-phenyl-hexahydro-pyrrolo[1,2-a][1,4]diazepine-1,7-dione **8a** as a 58:42 mixture of diastereoisomers (oily material). - IR (KBr): $\tilde{\nu}$ = 3365 cm^-1^ (m, br), 2925 (m), 1790 (w), 1733 (w), 1665 (vs, C=O), 1601 (m), 1491 (m), 1414 (m), 1249 (m), 1173 (m), 1070 (w), 864 (w), 738 (w), 697 (w). ^1^H NMR (CDCl_3_, 500 MHz): diastereoisomer A: δ = 2.18 ppm (m, 1H), 2.28-2.33 (m, 1H), 2.57 (m, 1H), 2.86 (m, 1H), 3.10 (dd, J = 14.5, 2.4 Hz, 1H), 3.86 (ddd, J= 15.4, 6.6, 1.5 Hz, 1H), 4.06 (dd, J = 15.4, 0.9 Hz, 1H), 4.11 (m, 1H), 4.38 (dd, J = 8.1, 1.8 Hz, 1H), 4.44-4.47 (m, 1H), 7.18 (m, 1H), 7.26 (m, 1H), 7.32-7.39 (m, 3H); diastereoisomer B: δ = 2.18 ppm (m, 1H), 2.28-2.33 (m, 1H), 2.57 (m, 1H), 2.83 (m, 2H), 2.86 (m, 1H), 3.75 (dd, J = 12.9, 1.6 Hz, 1H), 3.87 (m, 2H), 4.49 (m, 1H), 4.62 (m, 1H), 7.18 (m, 1H), 7.26 (m, 1H), 7.32-7.39 (m, 3H). ^13^C NMR (CDCl_3_, 125.8 MHz): diastereoisomer A: δ = 22.31 ppm (t), 29.86 (t), 49.34 (t), 54.69 (t), 61.54 (d), 64.95 (d), 126.94 (d), 129.28 (d), 144.73 (s), 169.65 (s, C=O), 176.42 (s, C=O); diastereoisomer B: δ = 22.08 ppm (t), 29.69 (t), 48.61 (t), 57.16 (t), 61.20 (d), 65.98 (d), 127.11 (d), 129.48 (d), 143.98 (s), 170.06 (s, C=O), 175.13. (s, C=O). MS (EI, 70 eV, 140 °C): *m/z* (%) = 260 (4) [M+], 176 (9), 167 (6), 149 (13), 120 (7), 106 (27), 98 (10), 84 (100), 83 (6), 71 (10), 69 (8), 57 (15), 56 (10), 55 (10), 43 (10), 41 (36). HRMS: calcd for C_14_H_16_N_2_O_3_: 260.11609; found 260.115938. **3^rd^ Fraction** (silica/ethanol; R_f_ = 0.46): 112 mg (60%) of 3-phenylaminomethyl-tetrahydro-pyrrolo[2,1-c][1,4]oxazine-1,6-dione **9a** (single diastereoisomer, *cis*-configuration according to calculations) as a colourless solid with mp 138 °C. - IR (KBr): $\tilde{\nu}$ = 3407 cm^-1^ (s, br), 2925 (m), 1790 (w), 1733 (w), 1665 (vs, C=O), 1601 (m), 1491 (m), 1414 (m), 1249 (m), 1173 (m), 1070 (w), 864 (w), 738 (w), 697 (w). ^1^H NMR (DMSO-D_6_, 500 MHz): δ = 1.87-1.91 ppm (m, 1H), 2.08-2.21 (m, 3H), 2.89 (m, 1H), 2.97 (m, 1H), 3.17 (dd, J = 13.7, 7.9 Hz, 1H), 3.27 (dd, J = 13.7, 3.1 Hz, 1H), 3.88 (m, 1H), 3.96 (m, 1H), 5.36 (s, br, 1H, NH), 6.50 (t, J = 7.3 Hz, 1H), 6.57 (m, 2 H), 7.04 ("t", "J" = 7.4 Hz, 2H). ^13^C NMR (DMSO-D_6_, 125.8 MHz): δ = 24.11 ppm (t), 30.06 (t), 47.62 (t), 48.03 (t), 63.79 (d), 66.57 (t), 112.17 (d), 115.55 (d), 128.82 (d), 149.04 (s), 175.40 (s, C=O), 175.92 (s, C=O). MS (EI, 70 eV, 215 °C): *m/z* (%) = 261 (2), 260 (14) [M+], 130 (7),107 (10), 106 (100), 104 (6), 93 (6), 84 (11), 77 (14), 44 (23), 41 (7). HRMS: calcd for C_14_H_16_N_2_O_3_: 260.11609; found 260.11568.

**Base-induced cyclization of 7b (Table 1, entry 4):** 207 mg (0.646 mmol) of **7b** in 20 ml of THF were treated with 26 mg (0.65 mmol) of sodium hydride (60% in mineral oil) for 1.5 h at room temperature under nitrogen. After addition of 1 equiv. of NaHPO_4_/water the mixture was concentrated at 50 °C/<1 mbar. The residue was fractionated by flash chromatography (silica, ethyl acetate as first eluent, ethanol as second). **1^st^ Fraction** (silica/ethyl acetate; R_f_ = 0.12): 74 mg (42%) of 4-hydroxy-2-(4-methylphenyl)-hexahydro-pyrrolo[1,2-a][1,4]diazepine-1,7-dione **8b** as a 30:70 mixture of diastereoisomers (oily material). - IR (KBr): $\tilde{\nu}$ = 3362 cm^-1^ (m, br), 2926 (w), 2298 (w), 1662 (s, C=O), 1511 (m), 1416 (m), 1251 (m), 1178 (w), 1107 (w), 813 (w), 750 (w). ^1^H NMR (CDCl_3_, 500 MHz): diastereoisomer A: δ = 2.14 ppm (m, 1H), 2.27 (m, 1H), 2.32 (s, 3H), 2.54 (m, 1H), 2.83 (m, 2H), 3.09 ("d", "*J*" = 14.2 Hz, 1H), 3.83 (dd, *J =* 15.3, 6.6 Hz, 1H), 4.03 ("d", "*J*" *=* 15.2 Hz, 1H), 4.21 (m, 1H), 4.38 ("d", "*J*" = 8.0 Hz, 1H), 4.46 (m, 1H), 7.05 ("d", "*J*" = 8.3 Hz, 2 H), 7.18 ("d", "*J*" = 8.5 Hz, 2 H); diastereoisomer B: δ = 2.14 ppm (m, 1H), 2.27 (m, 1H), 2.34 (s, 3H), 2.54 (m, 1H), 2.83 (m, 2H), 3.71 ("d", "*J*" = 14.3 Hz, 1H), 3.93 (m, 2H), 4.44 (dd, *J=* 8.2, 1.7 Hz, 1H), 4.57 (m, 1H), 7.05 ("d", "*J*" = 8.3 Hz, 2H), 7.18 ("d", "*J*" = 8.5 Hz, 2H). ^13^C NMR (CDCl_3_, 125.8 MHz): diastereoisomer A: δ = 21.03 ppm (q), 24.79 (t), 29.89 (t), 49.36 (t), 57.75 (t), 61.68 (d), 65.11 (d), 126.51 (d), 129.74 (d), 136.82 (s), 142.15 (s), 169.76 (s, C=O), 176.52 (s, C=O); diastereoisomer B: δ = 21.03 ppm (q), 22.14 (t), 29.72 (t), 48.66 (t), 51.24 (t), 61.25 (d), 66.09 (d), 125.80 (d), 129.95 (d), 137.06 (s), 141.42 (s), 170.09 (s, C=O), 175.20 (s, C=O). MS (EI, 70 eV, 160 °C): *m/z* (%) = 274 (21) [M+], 210 (12), 171 (13), 158 (32), 142 (11), 140 (16), 126 (11), 124 (10), 121 (12), 120 (100), 98 (43), 91 (18), 84 (60), 73 (15), 56 (10), 55 (10), 44 (11), 42 (13), 41 (40), 39 (10). HRMS: calcd for C_15_H_18_N_2_O_3_: 274.13174; found 274.13132. **2^nd^ Fraction** (silica/ethanol; R_f_ = 0.39): 64 mg (36%) of 3-(4-methylphenylamino)methyl-tetrahydro-pyrrolo[2,1-c][1,4]oxazine-1,6-dione **9b** (single diastereoisomer, *cis*-configuration according to calculations) as a colourless solid with mp 158-163 °C. - IR (KBr): $\tilde{\nu}$ = 3425 cm^-1^ (s, br), 2925 (m), 1653 (s, C=O), 1515 (m), 1459 (m), 1417 (m), 1303 (m), 1183 (w), 1107 (w), 812 (m), 669 (w). ^1^H NMR (DMSO-D_6_, 500 MHz): δ = 1.89-1.92 ppm (m, 1H), 2.05-2.15 (m, 3H), 2.13 (s, 3H), 2.87 (dd, *J* = 12.6, 6.5 Hz, 1H), 2.94 (dd, *J* = 12.7, 5.4 Hz, 1H), 3.24 (m, 2H), 3.93 (m, 2H), 5.15 (s, br, 1H, NH), 6.48 (d, *J=* 8.3 Hz, 2H), 6.86 *(*d, *J =* 8.4 Hz, 2H). ^13^C NMR (DMSO-D_6_, 125.8 MHz): δ = 20.10 ppm (q), 23.91 (t), 30.01 (t), 47.41 (t), 47.88 (t), 63.37 (d), 66.61 (d), 112.37 (d), 112.97 (s), 129.30 (d), 146.80 (s), 150.67 (s), 175.43 (s, C=O), 176.68 (s, C=O). MS (EI, 70 eV, 210 °C): *m/z* (%) = 275 (4), 274 (21) [M+], 264 (5), 146 (4), 124 (4), 121 (10), 120 (100), 119 (4), 118 (7), 107 (9), 106 (7), 91 (12), 84 (9), 77 (4), 65 (6), 44 (5), 41 (6). HRMS: calcd for C_15_H_18_N_2_O_3_: 274.13174; found 274.13156.

**Base-induced cyclization of 7c (Table 1, entry 8):** 213 mg (0.633 mmol) of **7c** in 20 ml of DMF were treated with 26 mg (0.65 mmol) of sodium hydride (60% in mineral oil) for 1.5 h at room temperature under nitrogen. After addition of 1 equiv. of NaHPO_4_/water the mixture was concentrated at 50 °C/<1 mbar. The residue was fractionated by flash chromatography (silica, ethyl acetate as first eluent, ethanol as second). **1^st^ Fraction** (silica/ethyl acetate; R_f_ = 0.09): 46 mg (25%) of 4-hydroxy-2-(4-methoxyphenyl)-hexahydro-pyrrolo[1,2-a][1,4]diazepine-1,7-dione **8c** as a 42:58 mixture of diastereoisomers (oily material). - IR (KBr): $\tilde{\nu}$ = 3422 cm^-1^ (m, br), 2925 (w), 2341 (w), 1663 (s, C=O), 1509 (s), 1415 (m), 1297 (m), 1250 (m), 1178 (m), 1103 (w), 1030 (m), 834 (w). ^1^H NMR (CDCl_3_, 500 MHz): diastereoisomer A: δ = 2.16 ppm (m, 1H), 2.29 (m, 1H), 2.55 (m, 1H), 2.81 (m, 1H), 3.08 (dd, *J* = 14.5, 2.4 Hz, 1H), 3.78 (s, 3H), 3.82 (m, 1H) 4.03 ("d", *"J"* = 14.5 Hz, 1H), 4.11 (m, 1H), 4.37 (dd, *J=* 8.1, 1.7 Hz, 1H), 4.44 (m, 1H), 6.86 ("d", *"J"* = 9.1 Hz, 2H), 7.24 ("d", *"J"* = 9.1 Hz, 2H); diastereoisomer B: δ = 2.16 ppm (m, 1H), 2.29 (m, 1H), 2.55 (m, 1H), 2.81 (m, 2H), 3.70 ("dd", *"J"* = 14.4, 1.5 Hz, 1H), 3.79 (s, 3H), 3.92 (m, 2H), 4.44 (dd, *J=* 8.1, 1.8 Hz, 1H), 4.59 (m, 1H) 6.89 ("d", *"J"* = 9.1 Hz, 2H), 7.09 ("d", *"J"* = 9.1 Hz, 2H). ^13^C NMR (CDCl_3_, 125.8 MHz): diastereoisomer A: δ = 22.13 ppm (t), 29.73 (t), 48.62 (t), 55.50 (q), 57.40 (t), 61.22 (d), 66.02 (d), 114.53 (d), 127.16 (d), 136.91 (s), 158.32 (s), 169.84 (s, C=O), 175.19 (s, C=O); diastereoisomer B: δ = 22.13 ppm (t), 29.73 (t), 48.62 (t), 55.50 (q), 57.40 (t), 61.22 (d), 66.02 (d), 114.53 (d), 127.16 (d), 136.91 (s), 158.32 (s), 169.84 (s, C=O), 175.19 (s, C=O). MS (EI, 70 eV, 135 °C): *m/z* (%) = 290 (4) [M+], 149 (10), 136 (20), 114 (19), 107 (9), 106 (100), 105 (27), 104 (18), 100 (13), 84 (20), 79 (9), 78 (12), 77 (23), 72 (11), 63 (12), 58 (11), 51 (9), 45 (13), 43 (11), 41 (16). HRMS: calcd for C_15_H_18_N_2_O_4_: 290.12666; found 290.12591. **2^nd^ Fraction** (silica/ethanol; R_f_ = 0.37): 64 mg (36%) of 3-(4-methoxyphenylamino)methyl-tetrahydro-pyrrolo[2,1-c][1,4]oxazine-1,6-dione **9c** (single diastereoisomer, *cis*-configuration according to calculations) as a colourless solid with mp 134-137 °C. - IR (KBr): $\tilde{\nu}$ = 3443 cm^-1^ (s, br), 2335 (w), 1657 (s, C=O), 1591 (s), 1511 (s), 1462 (m), 1411 (m), 1299(m), 1237(m), 1182(w), 1107(w), 1031 (w). ^1^H NMR (DMSO-D_6_, 500 MHz): δ = 1.86-1.90 ppm (m, 1H), 2.02-2.22 (m, 3H), 2.83 (dd, *J* = 12.4, 6.6 Hz, 1H),2.92 (dd, *J* = 12.4, 4.9 Hz, 1H), 3.08 ("dd", "*J*" = 13.7, 8.5 Hz, 1H), 3.31 (dd, *J* = 13.9, 2.7 Hz, 1H), 3.63 (s, 3H), 3.83 (m, 1H), 3.97 (m, 1H), 4.93 (s, br, 1H, NH), 6.54 ("d", "*J*" = 8.9 Hz, 2H), 6.69 ("d", "*J*" = 8.9 Hz, 2H). ^13^C NMR (DMSO-D_6_, 125.8 MHz): δ = 24.23 ppm (t), 30.11 (t), 48.36 (t), 48.59 (t), 55.30 (q), 64.05 (d), 66.58 (d), 113.31 (d), 114.53 (d), 143.35 (s), 150.67 (s), 175.36 (s, C=O), 175.59 (s, C=O). MS (EI, 70 eV, 200 °C): *m/z* (%) = 2291 (8), 290 (30) [M+], 262 (10), 137 (9), 136 (100), 123 (10}, 108 (16), 85 (8), 84 (65), 45 (8), 44 (22), 43 (9), 41 (33). HRMS: calcd for C_15_H_18_N_2_O_4_: 290.12666; found 290.12619.
